# Brazilian Protocol for Sexually Transmitted Infections, 2020: sexually transmitted enteric infections

**DOI:** 10.1590/0037-8682-598-2020

**Published:** 2021-05-17

**Authors:** Edilbert Pelegrini Nahn, Eduardo Campos de Oliveira, Marcelo Joaquim Barbosa, Thereza Cristina de Souza Mareco, Helena Andrade Brígido

**Affiliations:** 1 Universidade Federal do Estado do Rio de Janeiro, Campus Macaé, RJ, Brasil; 2 Secretaria Estadual de Saúde de Santa Catarina, Florianópolis, SC, Brasil; 3 Secretaria de Saúde do Distrito Federal, Brasília, DF, Brasil; 4 Ministério da Saúde, Brasília, DF, Brasil; 5 Universidade Federal do Pará, Belém, PA, Brasil

**Keywords:** Sexually transmitted diseases, Diarrhea, Sexual behavior, Drug therapy

## Abstract

The sexually transmitted enteric infections topic is one of the chapters of the Clinical Protocol and Therapeutic Guidelines for Comprehensive Care for People with Sexually Transmitted Infections, published by the Brazilian Ministry of Health in 2020. The document was developed based on scientific evidence and validated in discussions with specialists. This article presents epidemiological and clinical aspects of these infections and guidance for service managers on their programmatic and operational management. The aim is to assist health professionals with screening, diagnosis, and treatment of people with sexually transmitted enteric infections and their sexual partners, in addition to supporting strategies for their surveillance, prevention, and control.

## Highlighted excerpt

The incidence of anorectal sexually transmitted infections has increased over the last years, mainly due to the increase in the practice of unprotected receptive anal sexual intercourse

## FOREWORD

This article addresses sexually transmitted enteric infections, a topic that comprises the Clinical Protocol and Therapeutic Guidelines (PDCT) for Comprehensive Care for People with Sexually Transmitted Infections (STI), published by the Health Surveillance Department of the Brazilian Ministry of Health. For elaborating the PDCT, a selection and analysis of the evidence available in the literature were performed, and a panel of specialists discussed it. The document was approved by the National Committee for the Incorporation of Technologies in the Brazilian National Health System (Conitec) and updated by the panel of specialists in STI in 2020^1^. 

## EPIDEMIOLOGICAL ASPECTS

Enteric pathogens and anorectal infections can be transmitted through different sexual practices without barrier protection in receptive anal or oroanal sex^2^. The transmission of diverse agents occurs naturally through the fecal-oral route, generally caused by consumption of contaminated food or water. Sexual transmission is well described, and it can happen through direct oroanal practice or indirectly through oral sex after anal penetration or through the use of fingers or fomites^3^. 

Anorectal STI incidence has augmented over the last years, mainly due to the increased frequency of unprotected receptive anal sexual intercourse^4^. Anorectal intercourse is common, although its exact frequency stays unknown due to asymptomatic infections and the lack of accurate epidemiological data. People with symptoms or anorectal lesions are usually referred to coloproctologists for assessment and management^5^.

Risks to health arising from anal sex seem to be significantly underestimated by sexually active men and women in North America, Latin America, Asia, Africa, and other regions. Among heterosexual people, the reported prevalence of using condoms are almost universally lower in anal sex than in vaginal sex^6^.

Outbreaks of sexually transmitted enteric infections in men with sex with men (MSM) show very similar characteristics. Generally, men report having multiple sexual partners^7^ ^-^ ^10^, attending places specifically for sexual encounters^11^ ^-^ ^12^or particular sexual parties and using recreational drugs, including chemsex, or "chemical sex", sexual practice using crystal methamphetamine, gamma hydroxybutyrate, gamma-butyrolactone, or mephedrone immediately before or during sex^8^ ^,^ ^10^. Using the internet^7^ or geospatial network applications^9^ ^-^ ^10^ for finding casual partners that facilitate riskier behaviors is also commonly described^13^. 

Among the p enteric infections related to sexual transmission in MSM, hepatitis A, shigellosis, intestinal protozoan infections, such as amoebiasis and giardiasis, and bacterial gastroenteritis caused by *Campylobacter spp*. can be cited^14^. Herpes simplex virus, HSV, and *Neisseria gonorrhea* are also etiological agents of anorectal infections transmitted through anal sexual intercourse^14^.

Hepatitis A is a generally self-limited acute infectious disease caused by the hepatitis A virus, transmitted through the fecal-oral route by ingesting contaminated food and water or intimate contact with an infected person^15^. Outbreaks associated with sexual transmission in MSM are described since 2016 by the European Centre for Disease Prevention and Control^16^ ^,^ ^17^. Symptoms after a four-week average incubation period are more common in adults and include fever, discomfort, nausea, anorexia, abdominal pain, and jaundice. Recurring hepatitis and acute hepatic insufficiency can also occur^18^. Shigellosis caused by the Gram-negative bacterium *Shigella spp.* is characterized by severe bacillary dysentery^19^ ^,^ ^20^. Regular sexual transmission outbreaks of *Shigella sonnei* and *Shigella flexneri* among MSM are reported since the 1970s^21^ ^,^ ^22^. Sexually transmitted shigellosis is linked to different behaviors, including using douche, recreational drug use, and fisting, a practice consisting of forearm or hand introduction in the partner's vagina or anus^8^ ^-^ ^10^ ^,^ ^11^.

Proctocolitis is associated with food or water-transmitted diseases, including *Shigella spp.* ^23^. It is characterized by watery or bleeding diarrhea, abdominal pain, tenesmus, and, sometimes, fever and discomfort from four to seven days^24^ ^,^ ^25^. Dissemination of multiresistant *Shigella spp.* infections through the sexual route have been reported^26^ ^-^ ^28^. *Shigella spp.* strains among MSM show increasing resistance to multiple drugs, mostly azithromycin and ciprofloxacin^29^ ^-^ ^33^. 

The foremost intestinal protozoan infections of interest within STI scope are giardiasis and amoebiasis. Annually millions of people develop these infections, but only 10% to 20% of the infected individuals become symptomatic. The risk of death is more remarkable for amoebiasis due to its invasive nature^34^. Such protozoan infections characteristically present higher prevalences in areas where sanitary conditions are inadequate, especially in Africa, in the Indian subcontinent, and parts of Central and South America. People who have traveled to developing countries are possible vectors^35^. Such infections are generally contracted through the fecal-oral route by ingesting contaminated water or food^36^. The higher incidence of *Entamoeba histolytica* enteritis among homosexual men seems attributed to direct oral-anal sexual practice^37^ ^,^ ^38^ or through sex toys or fellatio. It can denote high-risk behavior and multiple exposures^11^. Giardiasis underdiagnosis in this context is frequent due to the low suspicion of such transmission routes^39^ ^,^ ^40^. 

In giardiasis, the most common symptoms include diarrhea, oily stool, flatulence, and abdominal swelling^41^ ^,^ ^42^. There can be proctitis^43^. The average incubation period for giardiasis is one to two weeks, and the symptoms average three to ten weeks^44^. For amoebiasis, the wide specter of intestinal infection varies from asymptomatic to transitory intestinal inflammation up to fulminant colitis, including megacolon, peritonitis, and hepatic abscess^35^ ^,^ ^45^. The incubation period of intestinal amoebiasis is one to four weeks^46^. 

*Campylobacter spp*. is one of the most worldwide common causes of bacterial gastroenteritis^47^ ^,^ ^48^. Many outbreaks have been reported, including resistance to antimicrobial drugs such as ciprofloxacin and macrolides^49^, Extra-intestinal infection is rare. Still, it can lead to complications, including bacteremia, lung infection, meningitis, or reactive arthritis, mainly in immunocompromised people^47^ ^,^ ^48^. Ingestion of contaminated food and water and contact with pets are among the principal forms of transmission. There are reports of fecal-oral sexual transmission in places of sexual encounters with recreational drug use^50^ ^,^ _^51^_. 

HSV infections are characterized by chronicity and recurrence, with variable latency periods. There are two different strains: HSV-2, responsible for genital lesions, and HSV-1, for extragenital ones, specially orolabial^52^. However, it is possible to find inversions in such order, without clinical specter differences. HSV-1 is commonly acquired in childhood and adolescence, while HSV-2 is linked to sexual activity. The infection risk increases with the number of sexual partners throughout life^53^.

Gonorrhea is a common bacterial infection, transmitted almost exclusively through sexual or perinatal contact, affecting mainly the urethral and cervical mucous membranes and, less frequently, those in the rectum, oropharynx, and conjunctive^54^. Rectal *N. gonorrhoeae* infection is acquired through receptive anal intercourse and perineal contamination with cervicovaginal secretions. Around 35% of women with gonococcal cervicitis will present a concurrent rectal infection through infection contiguous dissemination^55^.

## CLINICAL ASPECTS

The presence of rectal bleeding and wounds or lesions in the anal and perianal area, possibly with pruritus and pain, producing secretions, indicate STI^23^.

Enteric pathogens cause gastroenteritis, which can have low (rectum) symptoms, such as pain, mucopurulent anal discharge, tenesmus and hematochezia, and high symptomatology (colon), such as diarrhea with a sudden start. When evolved with rectal distensibility loss, diarrhea becomes intense, and when the duodenum is compromised, vomits, and abdominal pains associated with colic occur^56^ ^,^ ^57^. 

In severe cases, significant morbidity and mortality can be associated with diarrhea, dehydration, bacteremia, hemolytic uremia, and Guillain-Barré syndrome^58^ ^-^ ^59^.

The most significant complications encompass inflammation of the rectal mucosa extending to the colon, with bleeding as a substantial sign, in addition to diarrhea, which leads to symptom intensification due to rectal distensibility loss. When the duodenum is compromised, vomits and abdominal pains associated with colic occur^56^. 

## DIAGNOSIS

Diagnosis based only on clinical aspects lacks specificity, requiring laboratory examination to identify the enteric infection etiological agent and define its sexual transmission.

Serological markers - IgM and anti-HAV IgG antibodies - are specific examinations for hepatitis A laboratory diagnosis. Leukopenia, aminotransferase, and high bilirubin findings are unspecific^60^. 

For identifying *Shigella spp.*, bacteria insulation is conducted in cultures, mainly in hemoculture and coproculture, in addition to sensitivity tests to antimicrobial drugs for following resistance and drug interaction possible cases^61^. 

Amoebiasis laboratory diagnosis is usually based on microscopic and serological methods, including enzyme-linked immunosorbent assay, ELISA, and indirect hemagglutination assay, latex agglutination, and tests based on nucleic acid amplification^62^. Intestinal amoebiasis diagnosis in many countries usually depends on fecal sampling microscopic examination regarding the presence or lack of *E. histolytica* and *Giardia lamblia*. The proportion of asymptomatic people infected with this protozoan is not clear^62^. The diagnosis must be confirmed by detecting *E. histolytica*-specific antigen in stool to distinguish it from other nonpathogenic amoebae. Serological tests can contribute to the diagnosis of invasive diseases, such as amoebiasis. However, their sensitivity can vary according to the disease's type and stage^63^. Eliminating cysts of *G. lamblia* can be intermittent and last for weeks. Therefore, many samples must be collected for diagnosis. Collecting three samples on different days ideally allows identifying cysts in more than 90% of the cases compared with 50% to 70% of those with a single sample. ELISA or antibody direct immunofluorescence can identify the parasite, with 88%-98% sensitivity and 87%-100% specificity^64^. Endoscopic methods with aspiration and duodenal biopsy may be needed in cases of diagnostic greater difficulty^64^ ^,^ ^65^. 

*Campylobacter spp.* diagnosis is performed through isolation of the organism from stool samples or rectal swabs using selective media before starting antibiotic treatment. The culture identifies the subtype and susceptibility to antimicrobial drugs. Rapid tests for such pathogens, including antigen tests and nucleic acid-based tests, are available in Brazil^65^. 

Diagnosis of herpes infection is based on the clinical aspect, especially if the condition is recurrent, and on laboratory examinations, such as viral culture, antigen detection, and polymerase chain reaction^53^. For diagnosis and laboratory investigation of gonorrhea in symptomatic cases, using a swab for anal culture, antibiogram, and molecular biology detection are recommended. On the other hand, for asymptomatic individuals with receptive anal practice without condom use, the recommendation is biannual follow-up through anal swab for detection through molecular biology, highlighting that culture is less sensitive than molecular biology techniques. Extragenital material samples, particularly anal and pharyngeal, and molecular biology tests must necessarily be validated for such collection sites^14^.

## TREATMENT

Treatment of this infection group requires, at first, the identification or suspicion of the etiological agent, and it must start as soon as possible, not only aiming at relieving the symptoms but likewise other STI, also reducing transmission risk to other persons. Treatment includes antibiotics and parasiticide, in addition to hydroelectrolytic and symptomatic support medications.

Unspecific treatment for hepatitis A is conducted with hydration and symptomatic methods. The vaccine is the most efficient way for preventing transmission, which can also be applied after exposure together with immunoglobulin in people presenting high-risk^66^. For prevention in sexual contact, using an oral condom is the indication^67^.

The primary treatment for non-complicated *Shigella spp.* is conducted with ciprofloxacin, including azithromycin and ceftriaxone as alternative therapies. Prevention is accomplished through washing hands and food for consumption, in addition to sexual practices with protective barriers^68^ ^-^ ^71^, People living with human immunodeficiency virus, HIV, may have more severe and long-lasting shigellosis, mainly with T-CD4+ lymphocyte counting less than 200 cells/mm³. Antimicrobial therapy may be extended for six weeks^72^. Meanwhile, changes in mucosae can be gateways for HIV^25^.

For amoebiasis and giardiasis treatment, using nitroimidazole compounds, such as metronidazole, tinidazole, and secnidazole, is the recommendation, with high cure proportions. Using such drugs is a contraindication for women in the first trimester of pregnancy, breastfeeding women, and people with neurological disorders^35^. Albendazole and nitazoxanide are antiparasitic medications with efficiency similar to metronidazole against giardiasis and can be used as an alternative, in daily doses, for five to three days, respectively^73^. For giardiasis, symptoms typically improve within five to seven days after starting treatment. In the case of chronic forms, improvement is slower. In case of diarrhea prolongation, it is possible to request a parasitological stool examination for excluding giardiasis persistence^74^ ^,^ ^75^. Complications include hypokalemia, undernutrition, growth delay, cognitive deficits, arthritis, myopathy, irritable bowel syndrome, and chronic fatigue^76^ ^,^ ^77^.

*Campylobacter spp.* infection is self-limited and mild. Treatment is conducted with oral or parenteral hydration, depending on the disease severity and dehydration degree. Avoiding agents inducing intestinal mortality, as they can prevent infection resolution, is needed^49^. Antibiotics must also be considered for high-risk cases, such as immunocompromised and older people, and in case of more severe cases, with fever, hematochezia, or intense abdominal pain^78^. Antibiotic resistance, particularly resistance to fluoroquinolone, increased sharply since the 1990s. Different outbreaks were reported, including with resistance to antimicrobial drugs such as ciprofloxacin and macrolides^48^. 

Treatment of HSV infection is based on using aciclovir and its derivatives valaciclovir and famciclovir, which present better absorption through oral route and bioavailability. Topical aciclovir or other antiviral is effective in reducing symptoms, and intravenous use of aciclovir is recommended for special situations, such as disseminated disease, meningoencephalitis pictures, and pneumonitis. In occurrence cases equal to or higher than six episodes per year, suppressive therapy is the recommendation. Suppressive treatment duration varies, but it is generally longer than six months^50^.

For confirming *N. gonorrhoeae* as the infectious agent, ceftriaxone associated with azithromycin is recommended^14^. Treatment can also be conducted with presumptive diagnosis based on anamnesis on the history of receptive anal sexual history without protection^79^. 

## SURVEILLANCE, PREVENTION, AND CONTROL

Through Consolidation Ordinance GM/MS no. 4, of September 28, 2017, all viral hepatitis became a compulsory notification matter^80^. However, hepatitis A has mandatory notification since 2003^81^. The required notification forms are Available from: the Ministry of Health's website^82^. Other STIs also have a compulsory report. Those that are not compulsory at the federal level can be included in the notification list of the Federal District, states, and municipalities, with free autonomy, surveillance, and control, as long as sanitary standards are observed.

The diseases addressed in this article are transmitted through unprotected sexual intercourse and contaminated water, and food ingestion. Therefore, in addition to condom regular use, the recommendation of not performing sexual actions that may facilitate direct contact with feces, basic prevention measures, and sanitation are crucial.

General basic preventive measures include frequently washing hands, especially when preparing food, before the meals and after going to the toilet; drinking filtrated and chlorinated water; washing fruit and vegetables, and not ingesting food suspected to be contaminated^83^ ^,^ ^84^. 

Sanitation is comprehended as infrastructure and operational facilities for drinking water provision and sewage and urban cleaning with solid residue management and draining and urban rainwater management, generating better sanitary conditions for the population. 

Vaccination against hepatitis A, which, according to the National Immunization Program, is recommended in a single dose for all children between 15 months and five years old, is also effective prevention. Coinfection with hepatitis A virus is frequent among HIV-infected MSM. Studies suggest that early vaccination against hepatitis A in people living with HIV may not provide reliable protection against infection development of such virus. Therefore, post-exposure prophylaxis, immunoglobulin application, and the monovalent vaccine may be considered in hepatitis A virus high-risk recent situations, regardless of prior vaccine situation^66^.

The cure control of sexually transmitted enteric infections occurs through clinical follow-up after specific treatment.
